# Risk Factors of Typhoid Infection in the Indonesian Archipelago

**DOI:** 10.1371/journal.pone.0155286

**Published:** 2016-06-09

**Authors:** Sandra Alba, Mirjam I. Bakker, Mochammad Hatta, Pauline F. D. Scheelbeek, Ressy Dwiyanti, Romi Usman, Andi R. Sultan, Muhammad Sabir, Nataniel Tandirogang, Masyhudi Amir, Yadi Yasir, Rob Pastoor, Stella van Beers, Henk L. Smits

**Affiliations:** 1 KIT Biomedical Research, Royal Tropical Institute (KIT), Amsterdam, The Netherlands; 2 Department of Medical Microbiology, Molecular Biology and Immunology Laboratory, Faculty of Medicine, Hasanuddin University, Makassar, South-Sulawesi, Indonesia; 3 Department Microbiology, Faculty of Medicine, Mulawarman University, Samarinda, East-Kalimantan, Indonesia; Indian Institute of Science, INDIA

## Abstract

**Background:**

Knowledge of risk factors and their relative importance in different settings is essential to develop effective health education material for the prevention of typhoid. In this study, we examine the effect of household level and individual behavioural risk factors on the risk of typhoid in three Indonesian islands (Sulawesi, Kalimantan and Papua) in the Eastern Indonesian archipelago encompassing rural, peri-urban and urban areas.

**Methods:**

We enrolled 933 patients above 10 years of age in a health facility-based case-control study between June 2010 and June 2011. Individuals suspected of typhoid were tested using the typhoid IgM lateral flow assay for the serodiagnosis of typhoid fever followed by blood culture testing. Cases and controls were defined post-recruitment: cases were individuals with a culture or serology positive result (n = 449); controls were individuals negative to both serology and culture, with or without a diagnosis other than typhoid (n = 484). Logistic regression was used to examine the effect of household level and individual level behavioural risk factors and we calculated the population attributable fraction (PAF) of removing each risk significant independent behavioural risk factor.

**Results:**

Washing hands at critical moments of the day and washing hands with soap were strong independent protective factors for typhoid (OR = 0.38 95% CI 0.25 to 0.58 for each unit increase in hand washing frequency score with values between 0 = Never and 3 = Always; OR = 3.16 95% CI = 2.09 to 4.79 comparing washing hands with soap sometimes/never vs. often). These effects were independent of levels of access to water and sanitation. Up to two thirds of cases could be prevented by compliance to these practices (hand washing PAF = 66.8 95% CI 61.4 to 71.5; use of soap PAF = 61.9 95%CI 56.7 to 66.5). Eating food out in food stalls or restaurant was an important risk factor (OR = 6.9 95%CI 4.41 to 10.8 for every unit increase in frequency score).

**Conclusions:**

Major gains could potentially be achieved in reducing the incidence of typhoid by ensuring adherence to adequate hand-washing practices alone. This confirms that there is a pivotal role for ‘software’ related interventions to encourage behavior change and create demand for goods and services, alongside development of water and sanitation infrastructure.

## Introduction

Typhoid fever is an acute and often life-threatening febrile illnesses transmitted via the fecal-oral route by the bacterium *Salmonella enterica* serotype Typhi. Individuals are infected by ingestion of contaminated water or food but also after contact with a patient or ex-patient. Patients may become life-long carriers and these individual secreting the pathogen with their stool form a significant factor in the maintenance of the pathogen in a population. The pathogen does not infect animals, and therefore, transmission is only from human to human.

There is a lack of reliable data on the burden of typhoid, because of the limited availability of blood culture services and the challenges associated with implementing large scale fever surveillance techniques to measure disease incidence [1]. The latest estimates suggest that the disease caused 13.5 million illnesses globally in 2010 [2]. Worldwide, the highest incidence rates of typhoid fever have been recorded in Africa and Asia [2–3]. In Indonesia, a study conducted in the slums of Jakarta estimated the incidence rate of typhoid at 148.7 per 100000 person-years in the age group 2–4 years old, 180.3 in the age group 5–15 years old and 51.2 in those over 16 years of age, with a mean age of onset of 10.2 years [4]. Without effective treatment, typhoid fever has a case-fatality rate of 10–30%, but this number is reduced to 1–4% in those receiving appropriate therapy [1].

Historically the World Health Organisation has maintained the position that typhoid control should revolve around the treatment of acute cases combined with improvements in water and sanitation [1]. Two vaccines are licensed for use—the oral Ty21a vaccine and the injectable Typhoid polysaccharide vaccine [5]—in high-risk settings. Although positive experiences in Asia are paving the way for a renewed focus on vaccination programmes [6–7], reduced exposure to the disease by means of improved water and sanitation remains the cornerstone of typhoid prevention. However, there is a growing recognition that access to water and sanitation alone is not sufficient—until hygiene is properly practiced, both at home and in the community as a whole, the desired impact of improved water and sanitation services on health related outcomes (typhoid included) cannot be realized [8–9].

Knowledge of risk factors and the relative importance of access to water and sanitation (‘hardware’), versus individual behavior (‘software’) [10] is essential to develop effective health interventions. A variety of risk factors for typhoid fever have been reported in different studies in Indonesia and these can be generally divided in factors associated with low levels of education, contact with a typhoid patient, lack of access to clean water and sanitation, inadequate hand-washing practices and poor hygiene, as well as consumption of street food and drinks [11–14]. However, no study to date has systematically assessed the relative importance of ‘hardware’ vs. ‘software’ and across a variety of settings in Indonesia, encompassing both urban and rural areas. Furthermore it is not known how many cases could potentially be averted by reducing ‘high risk’ behavior without intervening on access to hardware. In this study, we examine the effect of household level and individual behavioural risk factors on the risk of typhoid and estimate the population attributable factor associated with the removal of main risk factors in three Indonesian islands encompassing rural, peri-urban and urban areas.

## Materials and Methods

### Study design and patient recruitment

**Fig 1:**
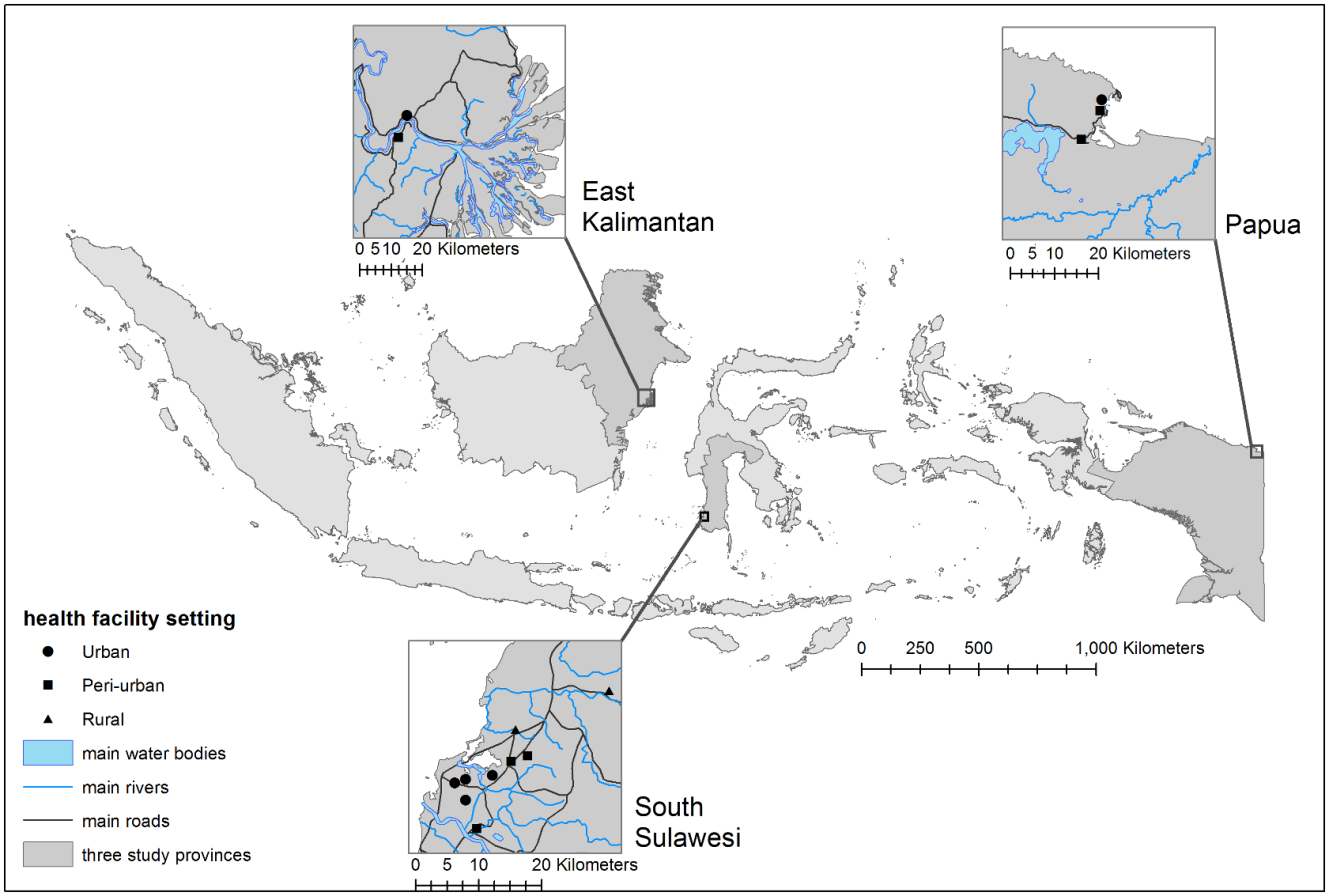
Location of study sites and participating health facilities.

A total of 933 patients were recruited in a case-control study between June 2010 and June 2011 from 14 selected hospitals and health centres in three Indonesian islands: in and around Makassar in South Sulawesi (Sulawesi); Jayapura (Papua); Samarinda in East-Kalimantan (Kalimantan) (Fig 1). Health facilities were located in a range of rural, urban and peri-urban settings—where peri-urban areas are defined as areas at the margins of an urban area, in transition from rural to urban. Health centres were included based on health staff availability, willingness to participate, as well as accessibility to a local diagnostic facility (to enable easy transport of blood culture material). This included two facilities in Kalimantan (one in a peri-urban area, one in an urban area), three in Papua (two in peri-urban areas and one in an urban area) and nine in Sulawesi (two in rural area, three in peri-urban areas and four in urban areas).

The criteria for enrolment in the study were the fulfillment of at least 2 of the following 3 typhoid suspicion criteria: 1) fever on the day of consultation (body temperature measured axillary >37.5°C); 2) fever duration ≥3 days; 3) headache since the start of the fever. Only individuals above the age of 10 years on the day of recruitment were included in the study.

**Fig 2:**
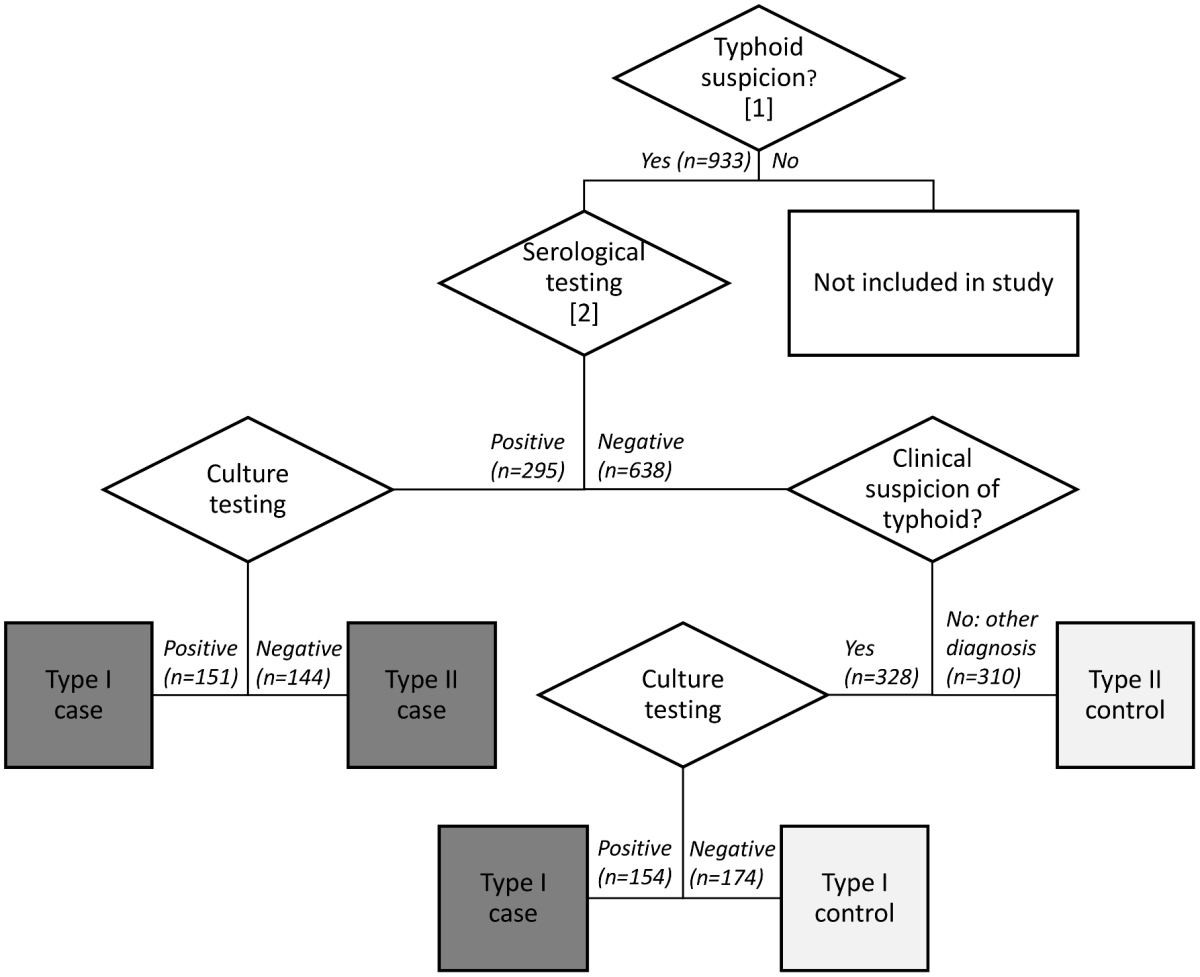
Diagnostic algorithm. ^1^ Fever on the day of consultation (body temperature measured axillary >37.5°C); fever duration ≥3 days; headache since the start of the fever. ^2^ IgM lateral flow assay

Individuals suspected of typhoid were tested by means of the typhoid IgM lateral flow assay for the serodiagnosis of typhoid fever followed by blood culture testing (see paragraph below for details). Three groups of approximately equal size were recruited: 1) patients who were positive to serology were enrolled as potential cases and a follow-up culture test was done; 2) patients who were negative to serology but for whom a clinical suspicion of typhoid remained, were enrolled as potential controls and were also tested by culture; 3) patients who were negative to serology and for whom there was no clinical suspicion of typhoid and were diagnosed with another disease were recruited as potential controls and no culture testing was done (Fig 2). This algorithm builds upon the complementarity between the lateral flow which, being an antibody test, has a higher sensitivity for later stage patients (from 7 days onward) and blood culture testing which has a high sensitivity for early stage patients. [15]. This algorithm was chosen in consultation with all staff involved in the study in order to accommodate 1) study goals (introduction of serological testing in remote health facilities where culture testing is not routinely conducted); 2) primary diagnostic concerns (a positive serology in a febrile patient with another diagnosis as typhoid may be due to sensitization in the past which warrants a follow-up of all serological test with culture testing); and 3) routine clinical practice, which prioritized the use of culture testing for negative serology only when no other diagnosis could be found—despite the fact that during the first week of the disease antibodies are often not detectable, which would justify a culture test for all negative serological tests.

The procedures for the typhoid IgM lateral flow assay which detects specific IgM antibodies have been documented elsewhere [15]. Test results were rated from 1+ to 4+ depending on the staining intensity at the test line and a result was considered positive when greater or equal to 1+. Blood cultures were performed by the inoculation of 8 ml freshly collected venopuncture blood directly into a Bactec plus aerobic/F bottle. Bacterial cultures were allowed to grow for 24 hours by incubation at 37°C after which 1 ml of broth was spread on a 9 cm diameter, 15 ml SS agar plate. After another incubation for 72 hours at 37°C plates were examined for colonies and individual colonies were picked and subject to biochemical identification.

Individual behavioural risk factors were collected at recruitment and included current and past illness, sanitary conditions and practices, personal hygiene including frequency of hand washing, (outdoor) eating habits and the use and preparation of safe drinking water, recent traveling, recent contact with a typhoid patients and knowledge of typhoid and the prevention of typhoid. In addition from December 2010 onwards a questionnaire was submitted to a household contact of each typhoid suspect to elicit information regarding household level access to water, hygiene and sanitation. Household level data was collected for a subset of 494 out of 933 individuals enrolled in the study.

Data collection was conducted by trained staff using semi-structured and pre-tested questionnaires. Information was collected on paper questionnaires and subsequently entered in an electronic database.

### Statistical analyses

For statistical analyses the following case definition was defined post-recruitment: Type I cases were individuals with a culture positive result regardless of serological result (c+/s+; c+/s-); Type II cases were individuals who had a culture negative but seropositive results (c-/s+); Type I controls were individuals negative to both serology and culture (s-/c-); Type II controls were individuals negative to serology and with a diagnosis other than typhoid (s-/d-). In this paper we conducted the main analyses comparing all controls (Type I and II) versus all cases (Type I and II). Sub-set analyses were conducted comparing only Type I cases and Type I controls.

The effect of individual and household level risk factors were examined by fitting logistic regressions. Since many individual risk factors were correlated, we built composite indicators to summarise the information from groups of collinear variables into a unique variable. An overall hand washing frequency score (hand washing in critical moments of the day) was created as the arithmetic mean of individual variables: hand washing after using the toilet, hand washing before eating, hand washing before food preparation, hand washing after change diapers, hand washing when coming home, hand washing before prayers. Each hand washing variable was coded at data collection on a categorical scale ranging from 0 (never) to 3 (always). The overall hand washing frequency score was therefore a continuous score variable ranging from 0 (never washes at critical times) to 3 (washes at all critical times). Similarly two frequency scores for eating meals at home or out were created out of individual categorical variables denoting the frequency with which breakfast, lunch and dinner were consumed at home or out (i.e. restaurant or food stall). This resulted in a continuous score between 0 (never consumes meals at home/out) to 3 (always meals at home/out).

We examined the population attributable fraction (PAF) of removing each individual behavioural risk factor, by island and by type of setting (urban, peri-urban and rural) to assess the proportion of cases which could be potentially averted by removing each factor. The PAF estimates and confidence intervals were estimated using the user defined punafcc command in Stata, which implements a maximum likelihood estimation of the attributable fraction from logistic models [16].

All analyses were conducted with Stata/SE 12.1 for Windows.

### Ethics

The medical ethical committee of the Hasanuddin University approved the study. Written informed consent was obtained from literate participants or their guardians if the participants were minors. Oral informed consent was obtained from all individuals enrolled in the study or their guardians. For illiterate participants who only provided oral consent, consent was documented with the signature of a witness. The use of oral consent for illiterate participants was specifically approved by the ethics committee.

## Results

Of the 933 patients in the study the following groups were recruited: 1) 295 individuals were positive to serology and enrolled as potential cases, 151 (51.2%) of which were tested positive by culture; 2) 328 individuals were negative to serology but with a clinical suspicion of typhoid and enrolled as potential controls, 154 (46.9%) of which were tested positive by culture; 3) 310 individuals were negative to serology and diagnosed with another disease and recruited as potential controls. Out of these 310 patients, 12 (3.9%) were diagnosed with dengue hemorrhagic fever, 37 (11.9%) with hepatitis, 48 (15.5%) with leptospirosis, 65 (21%) with malaria (21.0%), 66 (21.2%) with upper respiratory tract infection (21.3%) and 82 (26.5%) remained with an unknown diagnosis. According the categorical case definition defined post-recruitment for analyses, out of the 933 recruited patients, 305 (32.7%) were classified as Type I cases; 144 (15.4%) as Type II cases; 174 (18.6%) as Type I controls; and 310 (32.2%) as Type II controls. There was only one clear trend in socio-demographic characteristics across case definitions: cases were more likely to report a “food related” profession than controls (Table 1).

**Table 1:** Baseline characteristics by case definition.

	Typhoid case definition^1^									
	Type I Cases		Type II Cases		Type I Controls		Type II Controls		Total	
	N = 305		N = 144		N = 174		N = 310		N = 933	
**Setting**										
Urban	176	57.7%	78	54.2%	92	52.9%	169	54.5%	515	55.2%
Peri-urban	93	30.5%	55	38.2%	54	31.0%	99	31.9%	301	32.3%
Rural	36	11.8%	11	7.6%	28	16.1%	42	13.5%	117	12.5%
**Island**										
Kalimantan	77	25.2%	26	18.1%	53	30.5%	78	25.2%	234	25.1%
Papua	59	19.3%	34	23.6%	31	17.8%	63	20.3%	187	20.0%
Sulawesi	169	55.4%	84	58.3%	90	51.7%	169	54.5%	512	54.9%
**Seks**										
Male	144	47.2%	70	48.6%	82	47.1%	154	49.7%	450	48.2%
Female	161	52.8%	74	51.4%	92	52.9%	156	50.3%	483	51.8%
**Religion**										
Christian	44	14.4%	21	14.6%	26	14.9%	43	13.9%	134	14.4%
Hindu	6	2.0%	2	1.4%	2	1.1%	5	1.6%	15	1.6%
Muslim	255	83.6%	121	84.0%	146	83.9%	262	84.5%	784	84.0%
**Age category (in years)**										
> = 10–20	82	26.9%	27	18.8%	39	22.4%	71	22.9%	219	23.5%
> = 20–30	94	30.8%	45	31.3%	55	31.6%	92	29.7%	286	30.7%
> = 30–40	72	23.6%	35	24.3%	36	20.7%	61	19.7%	204	21.9%
> = 40–50	32	10.5%	22	15.3%	26	14.9%	44	14.2%	124	13.3%
> = 50	25	8.2%	15	10.4%	18	10.3%	42	13.5%	100	10.7%
**Profession**										
Low-skilled labour	46	15.1%	23	16.0%	32	18.4%	34	11.0%	135	14.5%
High-skilled labour	7	2.3%	17	11.8%	12	6.9%	15	4.8%	51	5.5%
Home based or unemployed	127	41.6%	67	46.5%	85	48.9%	144	46.5%	423	45.3%
Student	80	26.2%	29	20.1%	37	21.3%	84	27.1%	230	24.7%
Food related	14	4.6%	2	1.4%	1	0.6%	2	0.6%	19	2.0%
Farmer	31	10.2%	6	4.2%	7	4.0%	31	10.0%	75	8.0%
**Education**										
None	82	26.9%	28	19.4%	35	20.1%	72	23.2%	217	23.3%
Elementary	94	30.8%	51	35.4%	69	39.7%	94	30.3%	308	33.0%
Junior high	71	23.3%	45	31.3%	45	25.9%	77	24.8%	238	25.5%
Higher than junior high	58	19.0%	20	13.9%	25	14.4%	67	21.6%	170	18.2%

^1^ Type I controls were individuals negative to both serology and culture (s-/c-); Type II controls were individuals negative to serology and with a diagnosis other than typhoid (s-/d-); Type I cases were individuals with a culture positive result regardless of serological result (c+/s+; c+/s-); Type II cases were individuals who had a culture negative but seropositive results (c-/s+);

### Household characteristics

The distribution of household characteristics by case definition revealed that Type I controls differed from Type II controls and appear to be generally more similar to cases than controls (S1 Table)–they had worse access to water and sanitation, scored worse in terms of hygiene indicators (kitchen cleanliness, ownership of bins etc) and were from poorer households.

The strongest predictor of typhoid risk at household level was availability of soap near the toilet, increasing the odds over four-fold (S2 Table). This was closely followed by associations with variables related to sanitation, which increased the odds of typhoid between up to nearly threefold (methods to empty latrines, number of people sharing a latrine). Various variables related to water quality and accessibility were associated with the odds of typhoid, increasing odds by factors of around 2 (for example number of buckets available in the household for drinking, cooking and cleaning, availability of water near the latrine, water treatment, water colour, distance to nearest water point) as well as food preparation (number of home cooked meals per day, consumption of raw vegetables, number of times the kitchen is cleaned per week). Socio-economic variables were also associated with higher odds of typhoid in poorer households living in non-permanent or traditional houses without fridge or waste bins.

Household composition was not significantly (p>0.10) associated with the odds of typhoid (number of household members, number of household members under the age of 12). Although water availability (number of buckets available) and accessibility (distance to nearest water source, availability of water near the latrine) were predictors of typhoid risk, the type of water source (in-house tap, tap on compound, well on compound, well in village, rive/canal/pond) and storage containers (ember, drum, water tank), how water is taken from the place it is stored (scoop vs. tap) or whether water tasted of iron (correlated with water taste—yellow water more likely to taste like iron, water with no colour more likely to have no taste) were not shown to have an effect. Similarly, there was no effect of where food for household consumption is sourced (market, supermarket, stalls, peddlers). Although waste bin ownership was found to be a protective factor, whether the waste bin was covered, what kind of waste disposal was in place (burning, waste removal service, disposed in gutter/river, hole in the ground) did not have any effect. There was no effect with regards to reported flood in the last 12 months nor use of faeces as fertilizers (not reported by any participant).

Due to high collinearity in the variables from the household profile it was not possible to build a multivariate model to elicit independent risk factors of typhoid at household level.

### Individual behaviour

**Fig 3:**
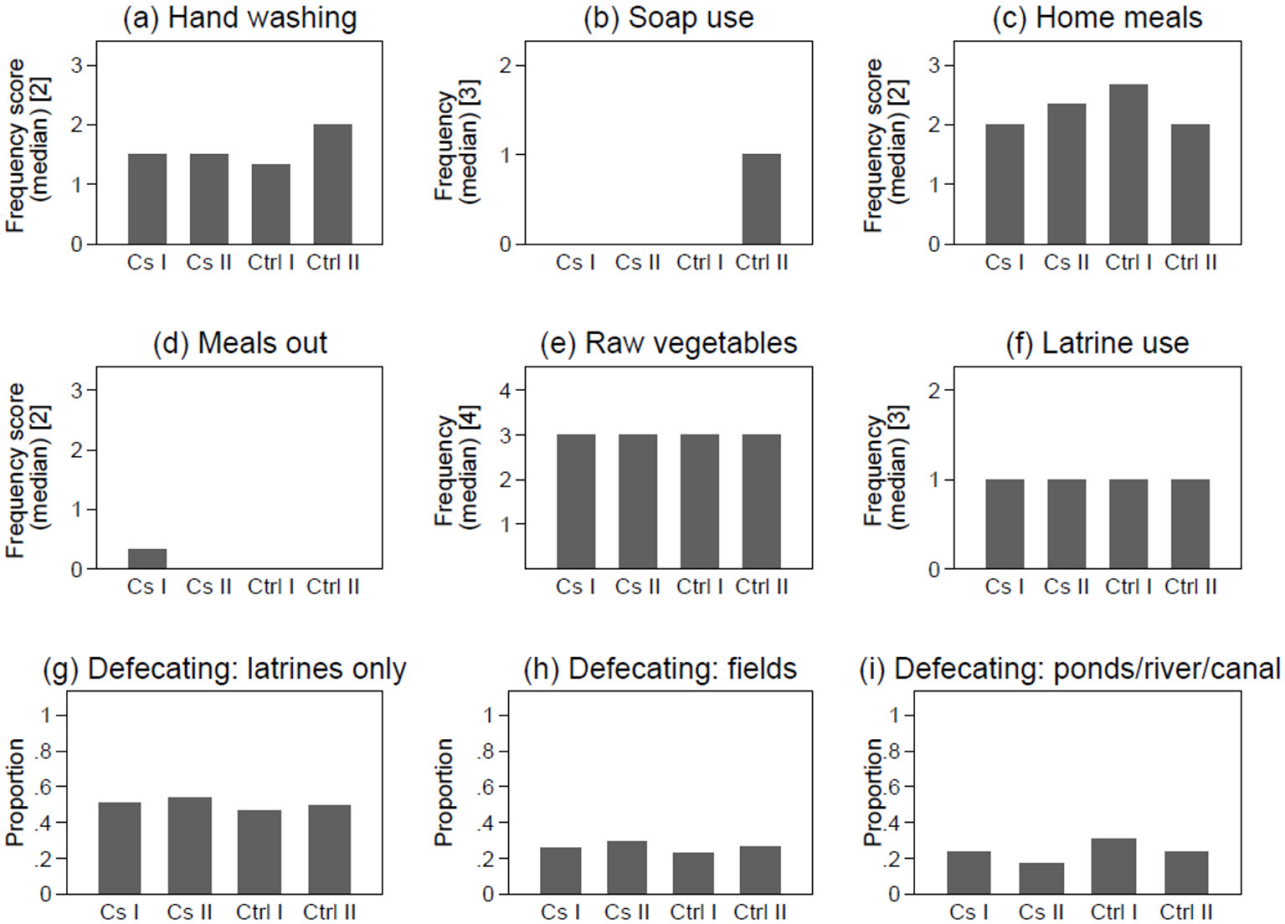
Distribution of individual level risk factors by case definition [1]. ^1^ Type I controls were individuals negative to both serology and culture (n = 305); Type II controls were individuals negative to serology and with a diagnosis other than typhoid (n = 144); Type I cases were individuals with a culture positive result regardless of serological result (n = 174); Type II cases were individuals who had a culture negative but seropositive results (n = 310); ^2^ Continuous score with values between 0 = Never and 3 = Always. ^3^ Categorical variable 0 = Sometimes/Never, 1 = Often, 2 = Always. ^4^ Categorical variable 1 = Less than once a week, 2 = Once a week, 3 = Few times a week, 4 = Every day

Graphical analyses and results from the logistic regression (both univariate and multivariate) revealed associations with only three behavioural variables: hand washing and washing with soap were strongly protective, whereas eating meals out was a strong risk factor as shown in Fig 3 and Table 2. Similarly to the household profile analyses reported above, analyses at individual level revealed that Type I controls differ in terms of behaviour from Type II controls, and are more similar to the cases—Type I controls have lower hand washing frequency and never report using soap.

**Table 2:** Estimated effect of individual level behavioural risk factors on the odds of typhoid^1^^,^^2^.

	Univariate				Multivariate (N = 933)		
	N	OR	95% CI	Sig.^3^	OR	95% CI	Sig.^3^
**Hand washing** frequency score^4^	933	0.26	0.20 to 0.35	<0.001	0.38	0.25 to 0.58	<0.001
**Use of soap**	933			<0.001			<0.001
Often		1			1		
Sometimes/Never		3.84	2.73 to 5.40	<0.001	3.16	2.09 to 4.79	<0.001
Always		0.61	0.37 to 0.99	0.049	0.60	0.33 to 1.07	0.083
**Eating meals at home** frequency score^4^	933	0.92	0.71 to 1.19	0.520			
**Eating meals out** frequency score^4^	933	2.30	1.63 to 3.24	<0.001	6.90	4.41 to 10.80	<0.001
**Raw vegetable consumption at home**	933			0.169			
Few times a week		1					
Every day		1.12	0.84 to 1.51	0.431			
Once/less a week		0.80	0.57 to 1.12	0.192			
**Latrine usage**	906			0.417			
Often		1					
Sometimes /Never		1.15	0.81 to 1.65	0.430			
Always		1.21	0.89 to 1.64	0.215			
**Places used to defecate**	933			0.925			
Only latrines		1					
Field		0.98	0.72 to 1.34	0.919			
Pond/river/canal		0.77	0.56 to 1.06	0.108			

^1^ Logistic regression comparing cases Type I and Type II (n = 449) to controls Type I and II (n = 484)

^2^ The effect of contact with a typhoid patient could not be estimated as the majority of patients (73%) did not know the answer to the question.

^3^ P-values reported: Wald test of significance of effect, LLR test of significance of variable in the model.

^4^ Continuous score with values between 0 = Never and 3 = Always

In the multivariate model, for every unit increase in the hand washing score there was an estimated 62% reduction in the odds of infection; people who reported to wash their hands with soap sometimes or never had threefold higher odds than those who washed their hands often, whereas there was no significant difference compared to those who reported to always wash their hands; eating meals out increased the odds of infection by a factor of 6.9. These effects from the final multivariate model did not change by more than 10% when adding the following significant hand washing related household level factors to the model (on the subset of individuals for whom both individual level and household level data was available): number of buckets of water available (n = 478), availability of soap near the latrine (n = 487), availability of a water source near the latrine (n = 487), and distance from the nearest water source (n = 492) (results not shown).

The sub-set analyses comparing Type I cases to Type I controls did not entirely confirm the main analyses. This is mainly because, as can be seen in S1 Table and Fig 3, and discussed above, Type I controls are more similar to Type I and Type II cases than to Type II controls in household characteristics and behavior. As a consequence effects were either less strong and/or no longer significant in the sub-set analyses (S3 Table), which means that the effects presented in the main analyses are likely to underestimate the true effects: Type I controls, which are actually more similar to cases, are aggregated together with other more genuine Type II controls thus diluting effects.

We calculated the PAF for the removal of each risk factor by type of setting (Table 3) and by island (Table 4) based on the univariate models (Table 2). Across all settings approximately two thirds of cases could potentially be averted if people washed their hands with soap often or always versus sometimes or never. Similarly approximately two thirds of cases could be averted with more frequent hand washing, i.e. if everyone washed their hands in all critical moments of the day often or always vs. sometimes or never. One in five cases could be averted by not eating meals out. The PAF associated with use of soap was especially high in rural areas where 92% of cases could be averted with use of soap, because the OR associated with soap use is much higher in this subgroup than in others (OR = 26.72, 95% CI = 6.00 to 118.83), whereas the prevalence of soap use is similar across settings (overall 39% of all individuals responded washing their hands with soap in all critical moments of the day). The PAF associated with eating meals out was the highest in Kalimantan and in peri-urban where estimated effects were higher than in other groups (Kalimantan: OR = 7.72 95% CI = 4.23 to 14.10; peri-urban areas: OR = 2.48 95% CI = 1.55 to 3.94) and where the practice is more prevalent (53.8% of individuals in Kalimantan and 45% of individuals in peri-urban areas reported eating meals out sometimes, often or always compared to 40% overall). The PAF associated with eating meals out in rural areas and in Sulawesi (where all the rural facilities were located) was close to zero and not significant.

**Table 3:** Population attributable fraction (PAF) and 95% CI of increased odds of typhoid infection for each behavioural risk factor, by type of setting^1^.

	Setting			
	Urban	Peri-urban	Rural	Total
	N = 515	N = 301	N = 117	N = 933
**Use of soap** ^2^	63.2	49.6	92.2	61.9
	(56.7 to 68.8)	(36.5 to 60.0)	(84.5 to 96.0)	(56.7 to 66.5)
**Hand washing** ^3^	65.0	74.1	53.8	66.8
	(57.5 to 71.1)	(66.1 to 80.3)	(23.1 to 72.2)	(61.4 to 71.5)
**Eating meals out** ^4^	15.8	33.8	-6.8	19.3
	(4.9 to 25.4)	(22.3 to 43.7)	(-4.0 to 18.7) ^5^	(11.7 to 26.3)

^1^ Odds ratios obtained from univariate logistic regression comparing cases Type I and Type II (n = 449) to controls Type I and II (n = 484)

^2^ Exposure: washing hands with soap often or always vs. sometimes or never

^3^ Exposure: washing hands in critical moments of the day average score > = 2 (often or always vs. sometimes or never)

^4^ Exposure: never eating out vs. eating out sometimes, often or always.

^5^ PAF is negative because in this subgroup eating meals out is a protective factor (OR = 0.81, 95%CI = 0.36 to 1.80). The Population Protective Factor (PPF) is 13.1% (95%CI -46.6% to 48.5%)

**Table 4:** Population attributable fraction (PAF) and 95% CI of increased odds of typhoid infection for each behavioural risk factor, by island ^1^.

	Island			
	Kalimantan	Papua	Sulawesi	Total
	N = 234	N = 187	N = 512	N = 933
**Use of soap** ^2^	61.5	53.7	66.9	61.9
	(48.2 to 71.3)	(45.7 to 60.7)	(59.6 to 72.9)	(56.7 to 66.5)
**Hand washing** ^3^	73.8	89.2	51.9	66.8
	(65.2 to 80.4)	(84.3 to 92.5)	(40.4 to 61.2)	(61.4 to 71.5)
**Eating meals out** ^4^	69.3	37.6	-4.5	19.3
	(62.4 to 74.9)	(15.9 to 53.7)	(-1.7 to 6.6) ^5^	(11.7 to 26.3)

^1^ Odds ratios obtained from univariate logistic regression comparing cases Type I and Type II (n = 449) to controls Type I and II (n = 484)

^2^ Exposure: washing hands with soap often or always vs. sometimes or never

^3^ Exposure: washing hands in critical moments of the day average score > = 2 (often or always vs. sometimes or never)

^4^ Exposure: never eating out vs. eating out sometimes, often or always.

^5^ PAF is negative because in this subgroup eating meals out is a protective factor (OR = 0.84 95%CI = 0.57 to 1.25). The Population Protective Factor (PPF) is 11.3% (95%CI -17.0% to 32.7%)

### Knowledge about prevention and transmission

The data regarding knowledge of typhoid transmission and its prevention was missing for 613 (65.6%) individuals who did not answer the corresponding questions for unclear reasons (no substantial differences across type of setting, island, sex, age, religion, although there were fewer missing values amongst the highly educated and those working in a ‘food related’ profession). Out of the 320 patients who answered, 176 (55.0%) reported that they did not know how it was transmitted, 102 (31.9%) mentioned ‘dirty environment’, 35 (10.9%) ‘lack of information/education’, 22 (6.9%) ‘contact with a patient’ and 2 (0.6%) ‘contaminated food’. Similarly, 175 (54.6%) reported that they did not know how typhoid could be prevented, 90 (28.1%) reported ‘cleaning the environment’, 51 (15.9%) ‘eating cooked food or drinking boiled/clean water’ 37 (11.6%) ‘washing hands before eating’, 13 (4.1%) ‘covering food’ or ‘storing food in containers’ and 7 (2.2%) avoiding contact with patients. The proportion who did not know about transmission and prevention was higher in peri-urban (22.9%) and rural (21.4%) areas compared to urban areas (15.9%) (chi-square p<0.001).

## Discussion

Our results suggest that major gains could be achieved in reducing the incidence of typhoid by ensuring adherence to adequate hand-washing practices—i.e. at critical moments of the day and with soap. These results confirm what has already shown extensively in the typhoid literature [11–14] as well as in the broader literature on disease transmitted by the fecal-oral route [17–19]. This is likely to extend to all other diarrheal diseases which remain an important public health problem in Indonesia [20] and the fourth most important cause of premature death [21].

Importantly, the effect of handwashing practices appears to be independent from household water availability and accessibility. We found associations between typhoid and with numerous household level variables related to availability and accessibility of water and soap, although we were not able to elicit independent effects—due to the high degree of collinearity between all risk factors we were not able to obtain a single stable multivariate model, rather, different methods (backward/forward manual elimination, automated stepwise, choice of variables by theme) produced greatly differing models which is why we refrained from presenting any of the results. However, when we refitted the final individual behavioral accounting for the potential confounding effect of household variables related to water availability and accessibility, estimated individual behavioural effects remained unchanged. This suggests that the estimated effects of hand washing practices in our study are independent from availability of water at household level.

These analyses confirm what has already been amply shown in the literature: sanitation hardware alone is ineffective as a tool to alleviate the burden of disease caused by disease transmitted by the fecal-oral route—what is needed is changes in behaviour coupled with improved access to sanitation. Statistics of access to water and sanitation in Indonesia have substantially improved in the past decade, and according to the latest Demographic and Health Survey in 2012 [22], three in four households in have access to an improved source of drinking water, and 68% of households have an improved toilet facility that is not shared with other households. Furthermore, most households (92%) have soap and water in the place where household members wash their hands, and only 6% have water only. However, health indicators show a prevalence of diarrhea in children under the age comparable to countries with much lower access to sanitation, with 14% of children under the age of five reporting diarrhea in the two weeks preceding the survey. On one hand it is possible that the level of ‘hardware’ sanitation currently present is still insufficient to contribute to improvement at the present level of environmental and food contamination due to a high number of carriers not having access to sufficient sanitation. These observations underscore the need for water and sanitation projects in Indonesia to keep focusing on ‘software’ components for hygiene promotion (to induce behavior change) and sanitation promotion (to create demand for sanitation and supply chains of goods and services) alongside infrastructural developments [9–10]. Indeed, hand-washing practices are probably more important when there is still a considerable contamination of water and food or a significant number of asymptomatic carriers in the community or in the environment of the patient.

Our population attributable factor (PAF) analyses enable a quantification of the number of cases which could be averted with more hygienic behavior: just over 60% of typhoid cases could potentially be averted if people washed their hands with soap nearly 67% could be averted if people washed their hands at all critical moments of the day, and 19% by not eating out (or more to the point—if hygienic practices in foodstalls were improved). Analyses of the PAF by type of setting and island did not show major differences in terms of the amount of cases which could potentially be averted with better hand washing practices. There were differences with regards to the number of cases which could potentially be averted if people did not eat out, which reflects the relative prevalence of this practice in different settings.

The main strength of our study is the wealth of data at individual and household level collected in three distinct Indonesian settings, which enabled to quantify the effect of individual factors, corrected for household level characteristics. Furthermore, since our sample covered three islands encompassing urban, peri-urban and rural settings, we were able to also investigate potential differences in the PAF of each independent risk factor across these different locations. However, these results should be interpreted with caution—due to our health facility selection strategy our estimates of the prevalence of hand washing and eating out are not necessarily representative of each island or of urban, peri-urban, and rural settings in Indonesia.

The main limitation of our study is the limited sensitivity of the tests and algorithm used. This means that both Type I and Type II controls are likely to contain a relatively high number of false negatives. In one study the sensitivity of the Typhoid IgM lateral flow was determined to be 62% and raises from 41% to almost 90% depending on the duration of illness compared to a sensitivity of 47% for blood culture [15]. In a study using a Bayesian model the sensitivity of the Typhoid IgM lateral flow assay was estimated to be 90% (compared to 81% sensitivity for blood culture) for children with a mean duration of illness of 5 days [23]. Conversely, specificity is much higher for both tests: whereas the specificity of blood culture is 100% the specificity of the Typhoid IgM lateral flow is reported to range between 85% [23] and 98% [15].

As a result, our study suffers from one of the main limitations of case-control studies, namely misclassification of the outcome variable which is likely to have diluted all the estimated effects. Following our algorithm, serological testing was done on all patients but culture was only done on individuals who were positive to serology (for confirmation) or for whom there was a clinical suspicion of typhoid (as a complimentary test to serology given its limited sensitivity for early stage patients). Therefore, there may be a number of false negatives in the Type II controls which could have ended up being Type I cases had a culture test been done. However, there are probably even more false negatives among the Type I controls given the limited sensitivity of both the Typhoid IgM lateral flow and blood culture testing used in this study for typhoid. There is also epidemiological evidence to suggest that this type of misclassification may indeed have occurred: the behaviour and household characteristics of Type I controls differs considerably from Type II controls—in fact, they are more similar to Type I and Type II cases. Nevertheless, one cannot exclude either that Type I controls were in fact infected with another enteric disease with similar symptoms to typhoid, given that health staff continued to suspect typhoid despite a negative serological test.

Despite these issues of potential differential misclassification of the outcome variable, it is worth pointing out that the risk of a differential misclassification of the risk factors, is likely to be low—the same tools were used for data collection in all cases and controls. Furthermore, the case definition used for analysis is different from the one used at recruitment (thus minimizing interviewer bias).

## Conclusions

Major gains could be achieved in reducing the incidence of typhoid by ensuring adherence to adequate hand-washing practices in Indonesia. This is likely to extend to all other diarrheal disease transmitted through the fecal-oral route, which remain an important public health problem in Indonesia. While not negating the paramount importance of investments in water supply and sanitation infrastructure, our results confirm that there is a pivotal role for ‘software’ related interventions to encourage behavior change and create demand for goods and services.

## Supporting Information

S1 Table Household (HH) characteristics by type of setting. ^1^ Type I controls were individuals negative to both serology and culture (s-/c-); Type II controls were individuals negative to serology and with a diagnosis other than typhoid (s-/d-); Type I cases were individuals with a culture positive result regardless of serological result (c+/s+; c+/s-); Type II cases were individuals who had a culture negative but seropositive results (c-/s+); ^2^ Data missing for 206 (41.7%) of respondents. ^3^ Based on US Dollar (USD) to Indonesian Rupiah (IDR) exchange rate on 31 December 2010: 1USD = 7470 IDR (www.exchangerates.org.uk)
(DOCX)

S2 Table Estimated effect of household level risk factors on the odds of typhoid (univariate analyses) ^1^. ^1^ Logistic regression comparing cases Type I and Type II (n = 235) to controls Type I and II (n = 259). ^2^ P-values reported: Wald test of significance of effect, LLR test of significance of variable in the model. ^3^ Based on US Dollar (USD) to Indonesian Rupiah (IDR) exchange rate on 31 December 2010: 1 USD = 7470 IDR (www.exchangerates.org.uk)
(DOCX)

S3 Table Estimated effect of individual level behavioural risk factors on the odds of typhoid—sub-set analyses ^1,2^. ^1^ Logistic regression comparing cases Type I (n = 305) to controls Type I (n = 174). The multivariate model was fitted using the same variables as the main analyses to allow cross comparisons between models. ^2^ The effect of contact with a typhoid patient could not be estimated as the majority of patients (73%) did not know the answer to the question.^3^ P-values reported: Wald test of significance of effect, LLR test of significance of variable in the model. ^4^ Continuous score with values between 0 = Never and 3 = Always
(DOCX)

## References

[1] Crump JA, Mintz ED. Global Trends in Typhoid and Paratyphoid Fever. Clin Infect Dis. 2010 1 15;50(2):241–6. 10.1086/649541 20014951 PMC2798017

[2] Buckle GC, Walker CLF, Black RE. Typhoid fever and paratyphoid fever: Systematic review to estimate global morbidity and mortality for 2010. J Glob Health. 2012 6;2(1):10401. 10.7189/jogh.02.010401 PMC3484760 23198130

[3] Crump JA, Luby SP, Mintz ED. The global burden of typhoid fever. Bull World Health Organ. 2004 5;82(5):346–53. 15298225 PMC2622843

[4] Ochiai RL, Acosta CJ, Danovaro-Holliday MC, Baiqing D, Bhattacharya SK, Agtini MD, et al A study of typhoid fever in five asian countries: disease burden and implications for controls. Bulletin of the World Health Organization. 2008 4;86(4):260–8. 18438514 10.2471/BLT.06.039818 PMC2647431

[5] Anwar E, Goldberg E, Fraser A, Acosta CJ, Paul M, Leibovici L. Vaccines for preventing typhoid fever. Cochrane Database Syst Rev. 2014;1:CD001261 10.1002/14651858.CD001261.pub3 24385413

[6] Ochiai RL, Acosta CJ, Agtini M, Bhattacharya SK, Bhutta ZA, Do CG, et al The Use of Typhoid Vaccines in Asia: The DOMI Experience. Clin InfectDis. 2007 7 15;45(Supplement 1):S34–8. 10.1086/518144 17582567

[7] DeRoeck D, Ochiai RL, Yang J, Anh DD, Alag V, Clemens JD. Typhoid vaccination: the Asian experience. Expert Rev Vaccines. 2008 7;7(5):547–60. 10.1586/14760584.7.5.547 18564010

[8] Peal Andy, Evans B, van der Voorder C. Hygiene and sanitation software—an overview of approaches. Water Supply & Sanitation Collaborative Council, Geneva; 2010.

[9] LaFond AK. A review of sanitation program evaluations in developing countries. Prepared jointly by UNICEF and EHP; 1995.

[10] Varley RC, Tarvid J, Chao DN. A reassessment of the cost-effectiveness of water and sanitation interventions in programmes for controlling childhood diarrhoea. Bull World Health Organ. 1998;76(6):617–31. 10191558 PMC2312499

[11] Sutiono AB, Qiantori A, Suwa H, Ohta T. Characteristics and risk factors for typhoid fever after the tsunami, earthquake and under normal conditions in Indonesia. BMC Res Notes. 2010;3:106 10.1186/1756-0500-3-106 20398409 PMC2873345

[12] Vollaard AM, Ali S, van Asten HAGH, Widjaja S, Visser LG, Surjadi C, et al Risk factors for typhoid and paratyphoid fever in Jakarta, Indonesia. JAMA. 2004 6 2;291(21):2607–15. 15173152 10.1001/jama.291.21.2607

[13] Gasem MH, Dolmans WM, Keuter MM, Djokomoeljanto RR. Poor food hygiene and housing as risk factors for typhoid fever in Semarang, Indonesia. Trop Med Int Health. 2001 6;6(6):484–90. 11422963 10.1046/j.1365-3156.2001.00734.x

[14] Velema JP, van Wijnen G, Bult P, van Naerssen T, Jota S. Typhoid fever in Ujung Pandang, Indonesia—high-risk groups and high-risk behaviours. Trop Med Int Health. 1997 11;2(11):1088–94. 9391512 10.1046/j.1365-3156.1997.d01-179.x

[15] Pastoor R, Hatta M, Abdoel TH, Smits HL. Simple, rapid, and affordable point-of-care test for the serodiagnosis of typhoid fever. Diagn Microbiol Infect Dis. 2008 6;61(2):129–34. 10.1016/j.diagmicrobio.2007.12.014 18276100

[16] Greenland S, Drescher K. Maximum likelihood estimation of the attributable fraction from logistic models. Biometrics. 1993 9;49(3):865–72. 8241375

[17] Curtis V, Cairncross S. Effect of washing hands with soap on diarrhoea risk in the community: a systematic review. Lancet Infect Dis. 2003 5;3(5):275–81. 12726975 10.1016/s1473-3099(03)00606-6

[18] Ejemot RI, Ehiri JE, Meremikwu MM, Critchley JA. Hand washing for preventing diarrhoea. Cochrane Database Syst Rev. 2008;(1):CD004265 10.1002/14651858.CD004265.pub2 18254044

[19] Cairncross S, Hunt C, Boisson S, Bostoen K, Curtis V, Fung ICH, et al Water, sanitation and hygiene for the prevention of diarrhoea. Int J Epidemiol. 2010 4;39 Suppl 1:i193–205. 10.1093/ije/dyq035 20348121 PMC2845874

[20] Agtini MD, Soeharno R, Lesmana M, Punjabi NH, Simanjuntak C, Wangsasaputra F, et al The burden of diarrhoea, shigellosis, and cholera in North Jakarta, Indonesia: findings from 24 months surveillance. BMC Infect Dis. 2005 10 20;5:89 16242013 10.1186/1471-2334-5-89 PMC1276796

[21] Institute for Health Metrics and Evaluation. Global Burden of Disease Country Profiles—GBD Profile: Indonesia. 2010.

[22] Statistics Indonesia (Badan Pusat Statistik—BPS), National Population and Family Planning Board (BKKBN), and, Kementerian Kesehatan (Kemenkes—MOH), and ICF International. Indonesia Demographic and Health Survey 2012. Jakarta, Indonesia; 2013.

[23] Moore CE, Pan-Ngum W, Wijedoru LPM, Sona S, Nga TVT, Duy PT, et al Evaluation of the Diagnostic Accuracy of a Typhoid IgM Flow Assay for the Diagnosis of Typhoid Fever in Cambodian Children Using a Bayesian Latent Class Model Assuming an Imperfect Gold Standard. Am J Trop Med Hyg. 2014 1 8;90(1):114–20. 10.4269/ajtmh.13-0384 24218407 PMC3886406

