# Supplementary material for: Risk Factors of Typhoid Infection in the Indonesian Archipelago
Source: PLoS One. 2016 Jun 9;11(6):e0155286. doi: 10.1371/journal.pone.0155286 (PMC4900629; doi:10.1371/journal.pone.0155286)
Supplement: S1 Table — 1 Type I controls were individuals negative to both serology and culture (s-/c-); Type II controls were individuals negative to serology and with a diagnosis other than typhoid (s-/d-); Type I cases were individuals with a culture positive result regardless of serological result (c+/s+; c+/s-); Type II cases were individuals who had a culture negative but seropositive results (c-/s+); 2 Data missing for 206 (41.7%) of respondents. 3 Based on US Dollar (USD) to Indonesian Rupiah (IDR) exchange rate on 31 December 2010: 1USD = 7470 IDR (www.exchangerates.org.uk) (DOCX) [file pone.0155286.s001.docx]

|  | **Typhoid case definiton** ^1^ | | | | | | | | | |
| --- | --- | --- | --- | --- | --- | --- | --- | --- | --- | --- |
|  | **Type I**  **Cases** | | **Type II**  **Cases** | | **Type I**  **Controls** | | **Type II**  **Controls** | | **Total** | |
|  | N=152 | | N=83 | | N=92 | | N=167 | | N=494 | |
| **Water availability** |  |  |  |  |  |  |  |  |  |  |
| <10 buckets | 65 | 44.2% | 32 | 41.0% | 40 | 46.0% | 38 | 22.9% | 175 | 36.6% |
| 10-15 buckets | 55 | 37.4% | 32 | 41.0% | 32 | 36.8% | 81 | 48.8% | 200 | 41.8% |
| >15 buckets | 27 | 18.4% | 14 | 17.9% | 15 | 17.2% | 47 | 28.3% | 103 | 21.5% |
| **Water treatment before drinking** |  |  |  |  |  |  |  |  |  |  |
| Always | 42 | 28.0% | 19 | 22.9% | 16 | 17.4% | 65 | 38.9% | 142 | 28.9% |
| Often | 17 | 11.3% | 11 | 13.3% | 13 | 14.1% | 31 | 18.6% | 72 | 14.6% |
| Sometimes | 54 | 36.0% | 37 | 44.6% | 36 | 39.1% | 56 | 33.5% | 183 | 37.2% |
| Never | 37 | 24.7% | 16 | 19.3% | 27 | 29.3% | 15 | 9.0% | 95 | 19.3% |
| **Water colour**: |  |  |  |  |  |  |  |  |  |  |
| No colour | 104 | 68.4% | 50 | 60.2% | 56 | 60.9% | 132 | 79.0% | 342 | 69.2% |
| Yellow | 48 | 31.6% | 33 | 39.8% | 36 | 39.1% | 35 | 21.0% | 152 | 30.8% |
| **Water distance:** |  |  |  |  |  |  |  |  |  |  |
| <5 min | 7 | 4.7% | 8 | 9.6% | 7 | 7.6% | 26 | 15.6% | 48 | 9.8% |
| 5-20 min | 143 | 95.3% | 75 | 90.4% | 85 | 92.4% | 141 | 84.4% | 444 | 90.2% |
| **Water source near latrine**  Yes | 131 | 86.2% | 73 | 88.0% | 77 | 83.7% | 166 | 99.4% | 447 | 90.5% |
| No | 16 | 10.5% | 10 | 12.% | 14 | 15.2% | 0 | 0% | 40 | 8.1% |
| **Soap near toilet** |  |  |  |  |  |  |  |  |  |  |
| Always | 13 | 8.8% | 6 | 7.2% | 11 | 12.1% | 36 | 21.7% | 66 | 13.6% |
| Often | 17 | 11.6% | 7 | 8.4% | 6 | 6.6% | 24 | 14.5% | 54 | 11.1% |
| Sometimes | 98 | 66.7% | 57 | 68.7% | 63 | 69.2% | 99 | 59.6% | 317 | 65.1% |
| Never | 19 | 12.9% | 13 | 15.7% | 11 | 12.1% | 7 | 4.2% | 50 | 10.3% |
| **Method empty latrine** |  |  |  |  |  |  |  |  |  |  |
| Desludge tank | 8 | 5.4% | 6 | 7.4% | 7 | 7.8% | 31 | 18.7% | 52 | 10.7% |
| Pit closed | 115 | 77.2% | 64 | 79.0% | 67 | 74.4% | 125 | 75.3% | 371 | 76.3% |
| Other | 26 | 17.4% | 11 | 13.6% | 16 | 17.8% | 10 | 6.0% | 63 | 13.0% |
| **Number of households who share the latrine** |  |  |  |  |  |  |  |  |  |  |
| Only household | 69 | 45.4% | 29 | 34.9% | 36 | 39.1% | 108 | 64.7% | 242 | 49.0% |
| 1-2 other households | 41 | 27.0% | 34 | 41.0% | 28 | 30.4% | 59 | 35.3% | 162 | 32.8% |
| 3-4 other households | 40 | 26.3% | 18 | 21.7% | 26 | 28.3% | 0 | 0.0% | 84 | 17.0% |
| >5 other households | 2 | 1.3% | 2 | 2.4% | 2 | 2.2% | 0 | 0.0% | 6 | 1.2% |
| **Home-cooked meals** |  |  |  |  |  |  |  |  |  |  |
| 2-3 per day | 117 | 77.0% | 62 | 74.7% | 67 | 72.8% | 165 | 98.8% | 411 | 83.2% |
| 1 per day | 35 | 23.0% | 21 | 25.3% | 25 | 27.2% | 2 | 1.2% | 83 | 16.8% |
| **Washing vegetables (for consumption raw)** ^2^ |  |  |  |  |  |  |  |  |  |  |
| Always | 8 | 8.5% | 2 | 4.5% | 1 | 1.8% | 19 | 20.4% | 30 | 10.4% |
| Often | 21 | 22.3% | 7 | 15.9% | 14 | 24.6% | 29 | 31.2% | 71 | 24.7% |
| Sometimes | 50 | 53.2% | 26 | 59.1% | 29 | 50.9% | 45 | 48.4% | 150 | 52.1% |
| Never | 15 | 16.0% | 9 | 20.5% | 13 | 22.8% | 0 | 0.0% | 37 | 12.8% |
| **Kitchen cleaning** ^2^ |  |  |  |  |  |  |  |  |  |  |
| Every day | 11 | 11.7% | 5 | 11.4% | 7 | 12.3% | 29 | 31.2% | 52 | 18.1% |
| 3 times a week | 19 | 20.2% | 5 | 11.4% | 7 | 12.3% | 20 | 21.5% | 51 | 17.7% |
| Once a week or less | 64 | 68.1% | 34 | 77.3% | 43 | 75.4% | 44 | 47.3% | 185 | 64.2% |
| **Flies in kitchen** ^2^ |  |  |  |  |  |  |  |  |  |  |
| Always | 63 | 67.0% | 32 | 72.7% | 44 | 77.2% | 43 | 46.2% | 182 | 63.2% |
| Often | 17 | 18.1% | 6 | 13.6% | 3 | 5.3% | 16 | 17.2% | 42 | 14.6% |
| Sometimes | 14 | 14.9% | 6 | 13.6% | 10 | 17.5% | 34 | 36.6% | 64 | 22.2% |
| **Fridge ownership** | 33 | 21.7% | 17 | 20.5% | 21 | 22.8% | 69 | 41.3% | 140 | 28.3% |
| **Wastebin ownership** | 110 | 72.4% | 61 | 73.5% | 58 | 63.0% | 154 | 92.2% | 383 | 77.5% |
| **Monthly HH income (USD)** ^3^ |  |  |  |  |  |  |  |  |  |  |
| 27 – 67 | 36 | 23.7% | 23 | 27.7% | 27 | 29.3% | 10 | 6.0% | 96 | 19.4% |
| 67 – 134 | 92 | 60.5% | 44 | 53.0% | 46 | 50.0% | 104 | 62.3% | 286 | 57.9% |
| > 134 | 24 | 15.8% | 16 | 19.3% | 19 | 20.7% | 53 | 31.7% | 112 | 22.7% |
| **Monthly HH food budget (USD)** ^2^ |  |  |  |  |  |  |  |  |  |  |
| 27 – 67 | 35 | 23.0% | 26 | 31.3% | 29 | 31.5% | 14 | 8.4% | 104 | 21.1% |
| 67 – 134 | 68 | 44.7% | 31 | 37.3% | 32 | 34.8% | 59 | 35.3% | 190 | 38.5% |
| > 134 | 49 | 32.2% | 26 | 31.3% | 31 | 33.7% | 94 | 56.3% | 200 | 40.5% |
| **Type of house** |  |  |  |  |  |  |  |  |  |  |
| Permanent | 26 | 17.1% | 12 | 14.5% | 16 | 17.4% | 52 | 31.1% | 106 | 21.5% |
| Semi-permanent | 105 | 69.1% | 58 | 69.9% | 59 | 64.1% | 115 | 68.9% | 337 | 68.2% |
| Non-permanent/traditional | 21 | 13.8% | 13 | 15.7% | 17 | 18.5% | 0 | 0.0% | 51 | 10.3% |
