# Supplementary material for: Risk Factors of Typhoid Infection in the Indonesian Archipelago
Source: PLoS One. 2016 Jun 9;11(6):e0155286. doi: 10.1371/journal.pone.0155286 (PMC4900629; doi:10.1371/journal.pone.0155286)
Supplement: S2 Table — 1 Logistic regression comparing cases Type I and Type II (n = 235) to controls Type I and II (n = 259). 2 P-values reported: Wald test of significance of effect, LLR test of significance of variable in the model. 3 Based on US Dollar (USD) to Indonesian Rupiah (IDR) exchange rate on 31 December 2010: 1 USD = 7470 IDR (www.exchangerates.org.uk) (DOCX) [file pone.0155286.s002.docx]

|  |  | | | |
| --- | --- | --- | --- | --- |
|  | N | OR | 95% CI | Sig.^2^ |
| **Water availability** | 478 |  |  | 0.017 |
| <10 buckets |  | 1 |  |  |
| 10-15 buckets |  | 0.62 | 0.41 to 0.93 | 0.021 |
| >15 buckets |  | 0.53 | 0.32 to 0.87 | 0.012 |
| **Water treatment before drinking** | 492 |  |  | 0.097 |
| Always |  | 1 |  |  |
| Often |  | 0.85 | 0.47 to 1.51 | 0.568 |
| Sometimes |  | 1.31 | 0.87 to 2.04 | 0.225 |
| Never |  | 1.68 | 0.99 to 2.82 | 0.053 |
| **Water colour**  yellow vs. no colour | 494 | 1.39 | 0.95 to 2.04 | 0.090 |
| **Water distance**  >5 mins vs. <5 mins | 492 | 2.12 | 1.12 to 4.02 | 0.021 |
| **Water source near latrine**  no vs. yes | 487 | 2.21 | 1.13 to 4.35 | 0.021 |
| **Soap near toilet** | 487 |  |  | 0.001 |
| Always |  | 1 |  |  |
| Often |  | 1.98 | 0.93 to 4.22 | 0.137 |
| Sometimes |  | 2.37 | 1.33 to 4.21 | 0.003 |
| Never |  | 4.40 | 2.00 to 9.65 | <0.001 |
| **Method empty latrine** | 486 |  |  | 0.002 |
| Desludge tank |  | 1 |  |  |
| Pit closed |  | 2.53 | 1.33 to 4.83 | 0.005 |
| Other |  | 3.86 | 1.75 to 8.53 | 0.001 |
| **Number of households who share the latrine** | 494 |  |  | <0.001 |
| Only household |  | 1 |  |  |
| 1-2 other households |  | 1.27 | 0.85 to 1.89 | 0.249 |
| 3-4 other households |  | 3.78 | 1.93 to 5.56 | <0.001 |
| >5 other households |  | 2.94 | 0.53 to 16.36 | 0.218 |
| **Home-cooked meals** 1 per day vs. 2-3 | 494 | 2.69 | 1.63 to 4.43 | <0.001 |
| **Washing vegetables for consumption raw** | 288 |  |  | 0.030 |
| Always |  | 1 |  |  |
| Often |  | 1.30 | 0.53 to 3.19 | 0.563 |
| Sometimes |  | 2.30 | 1.02 to 5.18 | 0.004 |
| **Kitchen cleaning** | 288 |  |  | 0.016 |
| Every day |  | 1 |  |  |
| 3 times a week |  | 2.00 | 0.89 to 4.48 | 0.092 |
| Once a week or less |  | 2.53 | 1.31 to 4.88 | 0.005 |
| **Flies in kitchen** | 288 |  |  | 0.009 |
| Always |  | 1 |  |  |
| Often |  | 1.11 | 0.56 to 2.17 | 0.764 |
| Sometimes |  | 0.41 | 0.23 to 0.76 | 0.004 |
| **Fridge ownership**  Not owning vs owning | 494 | 1.97 | 1.32 to 2.95 | <0.001 |
| **Waste bin ownership**  Not owning vs owning | 494 | 1.69 | 1.01 to 2.59 | 0.016 |
| **Monthly HH income (USD)** ^3^ | 494 |  |  | 0.001 |
| 27 – 67 |  | 1 |  |  |
| 67 – 134 |  | 0.57 | 0.35 to 0.91 | 0.019 |
| > 134 |  | 0.35 | 0.20 to 0.61 | <0.001 |
| **Monthly HH food budget (USD)** ^3^ | 494 |  |  | <0.001 |
| 27 – 67 |  | 1 |  |  |
| 67 – 134 |  | 0.77 | 0.47 to 1.24 | 0.282 |
| > 134 |  | 0.42 | 0.26 to 0.68 | <0.001 |
| **Type of house** | 494 |  |  | <0.001 |
| Permanent |  | 1 |  |  |
| Semi-permanent |  | 1.68 | 1.07 to 2.63 | 0.025 |
| Non-permanent/traditional |  | 3.58 | 1.77 to 7.24 | <0.001 |
