# Supplementary material for: Risk Factors of Typhoid Infection in the Indonesian Archipelago
Source: PLoS One. 2016 Jun 9;11(6):e0155286. doi: 10.1371/journal.pone.0155286 (PMC4900629; doi:10.1371/journal.pone.0155286)
Supplement: S3 Table — 1 Logistic regression comparing cases Type I (n = 305) to controls Type I (n = 174). The multivariate model was fitted using the same variables as the main analyses to allow cross comparisons between models. 2 The effect of contact with a typhoid patient could not be estimated as the majority of patients (73%) did not know the answer to the question.3 P-values reported: Wald test of significance of effect, LLR test of significance of variable in the model. 4 Continuous score with values between 0 = Never and 3 = Always (DOCX) [file pone.0155286.s003.docx]

|  | **Univariate** | | | | **Multivariate**  (N=479) | | |
| --- | --- | --- | --- | --- | --- | --- | --- |
| **Risk factors** | N | OR | 95% CI | Sig.^3^ | OR | 95% CI | Sig.^3^ |
| **Hand washing** frequency score ^4^ | 479 | 1.03 | 0.57 to 1.85 | 0.932 | 1.03 | 0.60 to 1.85 | 0.932 |
| **Use of soap** | 479 |  |  | 0.584 |  |  | 0.377 |
| Often |  | 1 |  |  | 1 |  |  |
| Sometimes/Never |  | 0.97 | 0.57 to 1.65 | 0.902 | 1.48 | 0.81 to 2.70 | 0.205 |
| Always |  | 1.49 | 0.58 to 3.86 | 0.410 | 1.72 | 0.63 to 4.70 | 0.292 |
| **Eating meals at home** frequency score  ^4^ | 479 | 0.34 | 0.22 to 0.53 | <0.001 |  |  |  |
| **Eating meals out** frequency score  ^4^ | 479 | 3.49 | 2.04 to 5.96 | <0.001 | 3.83 | 2.12 to 6.91 | <0.001 |
| **Raw vegetable consumption at home** | 479 |  |  | 0.394 |  |  |  |
| Few times a week |  | 1 |  |  |  |  |  |
| Every day |  | 0.94 | 0.61 to 1.45 | 0.781 |  |  |  |
| Once/less a week |  | 0.72 | 0.45 to 1.16 | 0.176 |  |  |  |
| **Latrine usage** | 468 |  |  | 0.347 |  |  |  |
| Often |  | 1 |  |  |  |  |  |
| Sometimes /Never |  | 0.94 | 0.56 to 1.58 | 0.824 |  |  |  |
| Always |  | 1.36 | 0.86 to 2.13 | 0.862 |  |  |  |
| **Places used to defecate** | 479 |  |  | 0.300 |  |  |  |
| Only latrines |  | 1 |  |  |  |  |  |
| Field |  | 1.01 | 0.63 to 1.61 | 0.958 |  |  |  |
| Pond/river/canal |  | 0.70 | 0.45 to 1.09 | 0.110 |  |  |  |
